# Avocado–Soybean Unsaponifiables Enhance Tendon Healing via Anti-Inflammatory and Antioxidant Mechanisms in a Rat Achilles Injury Model

**DOI:** 10.3390/medicina61112035

**Published:** 2025-11-14

**Authors:** Mustafa Dinç, Ömer Cevdet Soydemir, Hünkar Çağdaş Bayrak, Recep Karasu, Bilal Aykaç, Mehmet Emre Topcu

**Affiliations:** 1 Orthopedics and Traumatology Clinics, Bursa City Hospital, Bursa 16250, Turkey; dromer77@hotmail.com (Ö.C.S.); recepkarasu@hotmail.com (R.K.); draykac@gmail.com (B.A.); 2 Orthopedics and Traumatology Clinics, Çekirge State Hospital, Bursa 16090, Turkey; cagdasbayrak90@gmail.com; 3 Department of Pathology, Faculty of Veterinary Medicine, Bursa Uludag University, Bursa 16059, Turkey; mehmetemretopcu@uludag.edu.tr

**Keywords:** avocado–soybean unsaponifiables, tendon healing, Achilles tendon injury, oxidative stress, inflammation

## Abstract

*Background and Objectives:* Tendon healing is a multifactorial process influenced by inflammation and oxidative stress. Avocado–soybean unsaponifiables (ASU), recognized for their anti-inflammatory and antioxidant properties in osteoarthritis, have not yet been evaluated in tendon repair. This study aimed to investigate the effects of systemic ASU administration on histological, biomechanical, and biochemical parameters of tendon healing in a rat Achilles tendon injury model. *Materials and Methods:* Twenty male Wistar rats underwent bilateral Achilles tendon transection and repair. The ASU group received intraperitoneal ASU (300 mg/kg/day) for four weeks; controls received saline. Right tendons were analyzed histologically using a semiquantitative scoring system adapted from Curtis–DeLee, Bonar, and Modified Soslowsky criteria. Left tendons were tested biomechanically for maximum force, displacement, stress, stiffness, and energy parameters. Serum interleukin-1β (IL-1β), interleukin-6 (IL-6), tumor necrosis factor-α (TNF-α), total antioxidant status (TAS), total oxidant status (TOS), and oxidative stress index (OSI) were measured by ELISA. *Results:* ASU markedly improved histological healing with better collagen alignment, reduced inflammation, and normalized tenocyte morphology (*p* < 0.001). Biomechanical strength increased, with higher maximum force (*p* = 0.002), displacement (*p* = 0.004), stress (*p* = 0.001), and total energy to failure (*p* = 0.001). Serum IL-1β, IL-6, and TNF-α levels were lower (*p* < 0.001), while TAS increased and TOS/OSI decreased (*p* < 0.001). *Conclusions:* Systemic ASU administration enhances tendon healing by improving tissue organization, increasing mechanical strength, and modulating systemic inflammation and oxidative stress. These findings suggest that ASU may serve as a safe, clinically relevant adjunct therapy to promote tendon regeneration.

## 1. Introduction

Tendon injuries are common musculoskeletal disorders that result in pain, disability, and functional loss [1]. Despite the tendon’s limited intrinsic healing capacity, the biological response to injury proceeds through three overlapping phases: inflammation, proliferation, and remodeling [2,3]. However, this regenerative process is often inefficient and may result in fibrosis, disorganized collagen deposition, adhesion formation, or incomplete mechanical restoration [4,5].

In addition, chronic low-grade systemic inflammation—e.g., in obesity and type 2 diabetes—compromises tendon resilience; therefore, tendon-focused therapies may be more effective when implemented alongside strategies that attenuate chronic inflammation, including anti-inflammatory and antioxidant agents [6,7,8,9,10,11]. As such, there is growing interest in pharmacologic and biological approaches that enhance the quality and speed of tendon healing.

Inflammation plays a critical role in the early phase of tendon healing, but excessive or prolonged inflammatory activity may disrupt regeneration and promote degeneration [12,13]. Pro-inflammatory cytokines such as interleukin-1β (IL-1β), interleukin-6 (IL-6), and tumor necrosis factor-α (TNF-α) are markedly elevated after tendon injury and have been shown to impair tenocyte proliferation, inhibit collagen synthesis, and activate matrix-degrading enzymes such as matrix metalloproteinases (MMPs) [14,15]. Hence, modulation of the inflammatory cascade may preserve extracellular matrix integrity and improve tissue repair.

Oxidative stress is another pivotal factor influencing tendon healing. Reactive oxygen species (ROS), generated during inflammation and tissue injury, can induce mitochondrial dysfunction, lipid peroxidation, DNA damage, and tenocyte apoptosis [16,17]. Studies have demonstrated that the antioxidant defense system, including enzymatic components like superoxide dismutase (SOD) and glutathione peroxidase (GPx), plays a central role in protecting tendon tissue from oxidative damage [18,19,20]. Therefore, agents that mitigate oxidative stress may promote a more favorable healing microenvironment.

Avocado–soybean unsaponifiables (ASU), a natural compound extracted from avocado and soybean oils, has been widely studied in osteoarthritis for its anti-inflammatory, antioxidant, and chondroprotective properties [21,22]. It has demonstrated anti-inflammatory, antioxidant, and chondroprotective properties, primarily attributed to the downregulation of pro-inflammatory cytokines and the upregulation of antioxidant enzymes [23,24,25]. Among botanical unsaponifiable matrices, ASU is the most clinically characterized in musculoskeletal disease, supported by randomized trials and meta-analyses demonstrating symptomatic benefit in knee/hip osteoarthritis [26,27,28]. A further translational advantage is the availability of standardized, pharmaceutical-grade ASU formulations (ASU-E/Piasclédine 300; avocado: soybean 1:2 ratio) that support batch-to-batch consistency across studies [29,30]. In contrast, other botanical unsaponifiables (e.g., olive, rice bran, wheat germ) are chemically well described but lack comparable musculoskeletal clinical evidence, which is why we prioritized ASU to test whether OA-linked anti-inflammatory/antioxidant mechanisms translate into tendon repair [31,32,33]. However, its potential role in tendon healing remains unexplored.

Given the mechanistic parallels between cartilage and tendon degeneration—particularly the roles of inflammatory cytokines and oxidative stress—it is reasonable to hypothesize that ASU could enhance tendon healing through similar pathways. Therefore, this study aimed to evaluate the effects of systemic ASU administration on tendon healing in a rat Achilles tendon injury model. Histological, biomechanical, and biochemical analyses were performed to determine whether ASU treatment promotes tendon regeneration by modulating inflammation and oxidative stress. To our knowledge, this is the first study to investigate the therapeutic potential of ASU in tendon healing.

## 2. Materials and Methods

### 2.1. Study Design

This controlled experimental study was conducted in accordance with international guidelines for the care and use of laboratory animals and received ethical approval from the Institutional Animal Welfare Committee of the Medical Faculty of Uludağ University, Bursa, Turkey (Approval No: 2025-04/06, dated 4 March 2025). All procedures adhered to the principles outlined in the National Institutes of Health Guide for the Care and Use of Laboratory Animals. All animals used in this study were obtained from the Experimental Animals Breeding and Research Unit of the Medical Faculty of Uludağ University (Bursa, Türkiye). The animals were healthy, pathogen-free, and certified for use in biomedical research.

**Figure 1:**
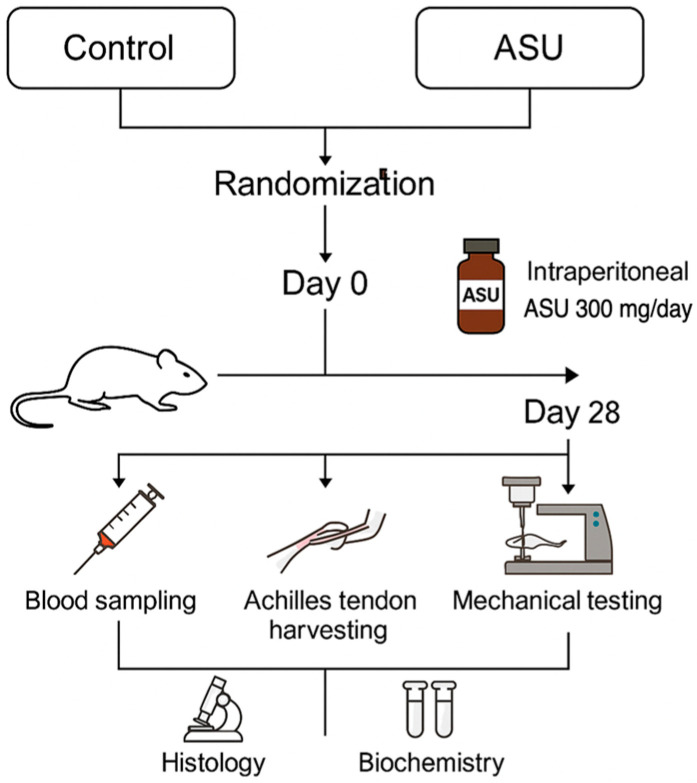
Study design timeline and sampling overview. Schematic representation of the experimental protocol for the rat Achilles tendon injury model. On Day 0, a complete tendon transection was performed under anesthesia. The ASU group received intraperitoneal ASU (300 mg/kg/day) for 28 days, while the control group received vehicle. On Day 28, all animals were euthanized under deep anesthesia. Blood samples were collected intraperitoneally, and Achilles tendons were harvested for histological, biochemical, and biomechanical analyses. The timeline summarizes key interventions and sampling points for both study groups.

Twenty adult male Wistar albino rats, aged 10 to 12 weeks and weighing between 300 and 350 g, were used in this study. Before the experimental procedures, all animals were allowed a 7-day acclimatization period in a controlled vivarium environment. They were housed in standard polycarbonate cages under optimal laboratory conditions, including a 12 h light/dark cycle, an ambient temperature of 22 ± 2 °C, and a relative humidity of 50–60%. All rats had unrestricted access to a standard pellet diet and tap water throughout the study period. After the acclimation phase, the animals were randomly assigned into two experimental groups (*n* = 10 per group) using a computer-generated randomization sequence: a Control group and an ASU treatment group. To minimize the number of animals used, a bilateral Achilles tendon injury model was employed [5,18,34,35]. Identical surgical procedures were performed on both hindlimbs. After the 28-day intervention period, right-sided tendons were allocated for histological analysis, and left-sided tendons for biomechanical testing. Environmental conditions, handling, and measurement procedures were standardized across all animals to minimize potential confounding factors. Group allocation was known only to the technician administering the treatments, while the investigators performing surgical procedures, histological evaluation, and statistical analysis were blinded to group identity. All animals that completed the 4-week postoperative period without surgical complications were included in the final analysis. No animals were excluded, and no predefined exclusion criteria were applied. All collected data points were included in the statistical analysis. The overall experimental timeline and sampling workflow are illustrated in Figure 1.

### 2.2. Surgical Procedure

**Figure 2:**
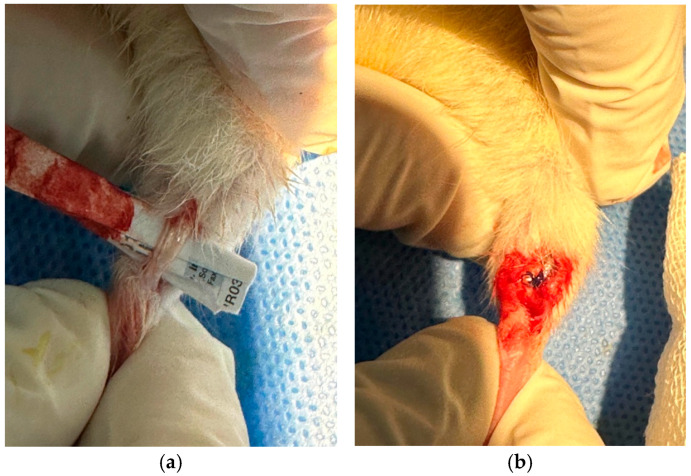
(**a**) Exposure of the Achilles tendon following surgical dissection. (**b**) Tendon ends are sutured after transection to complete the repair procedure.

All surgical procedures were conducted under sterile conditions in a designated laboratory operating room. General anesthesia was induced using 5% sevoflurane in an induction chamber and maintained with 2–3% sevoflurane via a nose cone during the operation. After confirming anesthesia depth through pedal reflex assessment, the hindlimbs of each rat was shaved and disinfected with 10% povidone-iodine solution. A longitudinal midline skin incision was made over the posterior aspect of each hindlimb to expose the Achilles tendon. The paratenon was carefully incised to expose the tendon fully. A complete transverse tenotomy was performed 5 mm proximal to the calcaneal insertion using microsurgical scissors. Hemostasis was achieved by gentle compression with sterile gauze. Each tendon was repaired using a modified Kessler suture technique with 5-0 non-absorbable polypropylene sutures, ensuring appropriate tension and alignment (Figure 2). Skin closure was completed with interrupted 4-0 nylon sutures. No postoperative immobilization or external fixation was applied, allowing free cage activity to simulate physiological loading. Animals were observed during recovery and monitored daily for signs of infection, wound dehiscence, or motor deficits.

### 2.3. Treatment Protocol

Avocado–soybean unsaponifiables (ASU) are industrially obtained from crude avocado and soybean oils via cold mechanical pressing, followed by saponification and molecular distillation to isolate the lipid unsaponifiable fractions while preserving thermolabile components [23]. In this study, we used a standardized formulation supplied by the manufacturer. Avocado–soybean unsaponifiables (ASU; Piasclédine 300, Expanscience Laboratories, Courbevoie, France) were provided as a powdered raw material containing a standardized 1:2 ratio of avocado and soybean unsaponifiable fractions. The powder was stored in airtight, light-protected containers at room temperature until use. Immediately before administration, the ASU powder was freshly suspended in sterile 0.9% saline to obtain a homogeneous suspension at a final concentration of 45 mg/mL. The suspension was vortexed for 1 min to ensure uniform dispersion and gently warmed to 37 °C to enhance solubility. Each rat received a daily intraperitoneal injection of 2 mL (≈90 mg/day, ≈300 mg/kg/day) starting from postoperative day 1 and continuing for 28 days. Fresh preparations were made daily and protected from light to minimize oxidative degradation of the lipid components. This dosage was selected based on previous preclinical studies that used comparable systemic doses to evaluate the anti-inflammatory and antioxidant effects of ASU in rodent models [36,37,38]. Control group animals received equal volumes (2 mL) of sterile saline via intraperitoneal injection over the same treatment period.

The intraperitoneal (i.p.) route was chosen to ensure accurate dosing and consistent systemic exposure. Compared with oral gavage, i.p. administration in rodents provides faster absorption, higher bioavailability, and reduced variability because it bypasses gastrointestinal degradation and first-pass hepatic metabolism. Pharmacokinetic comparisons have shown up to 6-fold higher absolute bioavailability and more rapid systemic distribution with the i.p. route compared with the oral route [39,40]. Moreover, oral or food-based supplementation in rodents is often confounded by variable gastric emptying, stress during gavage, and diet-dependent absorption of lipophilic compounds [41,42]. Given that ASU is a lipid-rich formulation susceptible to such variability, the i.p. route offered a reproducible means to achieve systemic delivery and uniform tissue exposure. Consistent with prior rat Achilles-tendon healing studies investigating antioxidant or anti-inflammatory compounds, we selected the i.p. route to maintain methodological comparability and reproducible pharmacologic exposure. Previous studies using caffeic acid, papaverine, L-carnitine, and erythropoietin all administered their agents intraperitoneally and demonstrated significant improvements in histological organization and biomechanical strength [43,44,45,46]. Similarly, other unsaponifiable lipid-based preparations, such as extra virgin olive oil and sesame oil fractions, have been delivered intraperitoneally in rodent models to ensure consistent systemic bioavailability and robust anti-inflammatory effects [47,48,49]. These precedents further support the i.p. route as an accepted and validated systemic administration method in experimental tendon-healing models.

### 2.4. Sample Collection and Processing

#### 2.4.1. Blood Sampling

At the end of the 28-day treatment period, all animals were anesthetized with 5% sevoflurane. Blood was collected via cardiac puncture into 5 mL syringes, placed in serum separator tubes, allowed to clot for 30 min at room temperature, and centrifuged at 3000 rpm for 10 min. Serum was aliquoted and stored at −80 °C until enzyme-linked immunosorbent assay (ELISA) analysis.

#### 2.4.2. Tendon Tissue Collection

After blood collection, rats were euthanized by cervical dislocation under deep anesthesia. Achilles tendons were dissected bilaterally from the musculotendinous junction to the calcaneal insertion. In each group, 10 right-sided tendons were fixed in 10% neutral-buffered formalin for histology, and 10 left-sided tendons were kept moist in saline-soaked gauze and subjected to biomechanical testing immediately after harvesting, without freezing.

### 2.5. Histological Analysis

Formalin-fixed tendons were immersed in 10% neutral-buffered formalin for 24–48 h, followed by dehydration through a graded ethanol series (70%, 80%, 90%, and 100%) and clearing in xylene. Samples were then embedded in paraffin blocks at 60 °C and sectioned longitudinally at 5 µm thickness using a rotary microtome (Leica RM2235, Leica Microsystems, Wetzlar, Germany) [50,51].

Sections were stained with hematoxylin and eosin (H&E) using a standardized protocol. Briefly, slides were deparaffinized in xylene, rehydrated through descending alcohol concentrations, and stained with Mayer’s hematoxylin for 5 min. After rinsing in running tap water for 5 min, slides were differentiated in 1% acid alcohol, blued in Scott’s tap water substitute, and counterstained with eosin Y (1%) for 2 min. Finally, slides were dehydrated, cleared in xylene, and mounted with a resinous medium [52]. All slides were examined under a light microscope (Olympus BX43, Tokyo, Japan) at ×100 and ×200 magnifications. For each tendon, at least three representative longitudinal sections were evaluated to minimize sampling bias.

Each section was scored using a combined semiquantitative histological scoring system that integrates validated criteria from Curtis & DeLee [53,54,55], Bonar [56,57,58], and the Modified Soslowsky [59,60,61] systems, enabling comprehensive assessment of both degenerative and reparative features of tendon healing.

The following seven parameters were evaluated: cartilage formation, tenocyte morphology, ground substance accumulation, inflammatory cell infiltration, neovascularization, fibroblastic activity, and collagen fiber alignment. Each domain was scored on an ordinal scale, and higher scores indicated more severe pathological alterations. The composite total score ranged from 0 (no abnormality) to 16 (severe degeneration).

All slides were independently evaluated by two board-certified pathologists, blinded to group allocation. In cases of discrepancy, a consensus score was reached through joint review. The scoring parameters and detailed criteria are summarized in Table 1.

### 2.6. Biomechanical Testing

**Figure 3:**
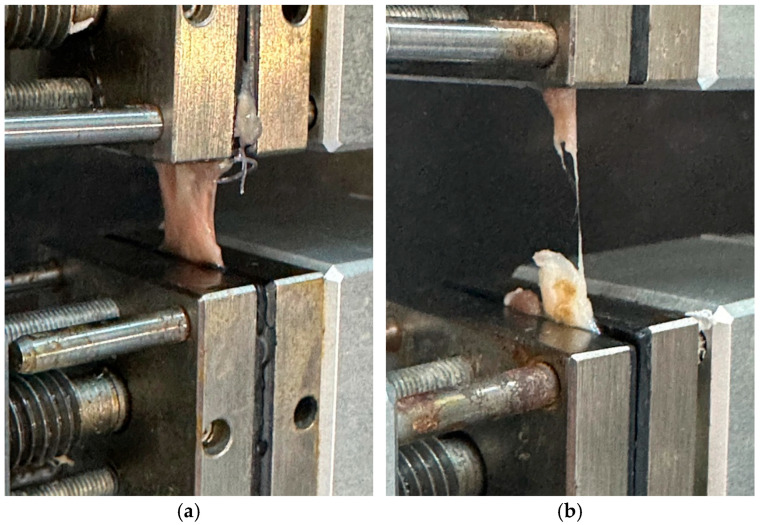
Tensile strength testing of rat Achilles tendon. Representative images of the biomechanical testing setup using a universal testing machine. The tendons were clamped and subjected to uniaxial tensile loading until failure. (**a**) Tendon positioned before testing. (**b**) Tendon at the point of rupture during load application.

Left-sided tendons (*n* = 10 per group) were kept moist in saline-soaked gauze and subjected to biomechanical testing immediately after harvesting, without prior freezing. All biomechanical tests were conducted using a universal testing machine (Shimadzu AG-X HS, Shimadzu Corporation, Kyoto, Japan) equipped with a 500 N load cell. Each tendon was mounted vertically between custom-designed clamps, ensuring secure fixation of the calcaneal and myotendinous ends to prevent slippage. During testing, the tendons were kept moist with saline spray. A uniaxial tensile load was applied at a constant displacement rate of 5 mm/min until complete rupture occurred (Figure 3). Load–displacement data were recorded continuously throughout the test. The following biomechanical parameters were recorded: maximum force, displacement at maximum force, stress, energy at maximum force, total energy to failure, and stiffness [34,43,62].

### 2.7. ELISA Analysis

Serum samples that had been stored at −80 °C were thawed only once before analysis to minimize degradation associated with repeated freeze–thaw cycles. Pro-inflammatory cytokines, including interleukin-1β (IL-1β), interleukin-6 (IL-6), and tumor necrosis factor-α (TNF-α), were quantified using rat-specific ELISA kits (Elabscience, Houston, TX, USA). Each measurement was performed in duplicate, and mean values were used for statistical analysis to reduce variability. Negative and positive controls supplied by the manufacturer were included on each plate to ensure assay validity, and standard curves were generated for every run.

Oxidative stress parameters—total antioxidant status (TAS) and total oxidant status (TOS)—were determined using ELISA kits (Rel Assay Diagnostics, Gaziantep, Turkey) based on the colorimetric methods described initially by Erel [63]. The TAS assay quantifies the ability of antioxidants in the sample to neutralize the ABTS^+^ radical cation, with results expressed as mmol Trolox equivalent/L/L. The TOS assay is based on the oxidation of ferrous ions to ferric ions by oxidants present in the sample, producing a colored complex with xylenol orange, and the results were expressed as μmol H_2_O_2_ equivalent/L/L. The oxidative stress index (OSI) was calculated as OSI = (TOS/TAS) × 100 after unit standardization. All measurements were performed in duplicate, and calibration curves were established for each run to ensure reproducibility.

### 2.8. Statistical Analysis

All statistical analyses were performed using IBM SPSS Statistics version 27.0 (IBM Corp., Armonk, NY, USA). The distribution of continuous variables was assessed using the Shapiro–Wilk test. Parameters that followed a normal distribution were compared using the independent samples *t*-test, whereas non-normally distributed variables were analyzed with the Mann–Whitney U test. Before the study, a power analysis was conducted using G*Power 3.1. Based on previously published studies with similar parameters, and in accordance with the resource equality model, it was estimated that including 10 animals per group (20 in total) under the assumption of equal variance would provide a statistical power exceeding 90%. All animals survived the procedure and completed the 4-week experimental period. No animals or data points were excluded from the analysis.

## 3. Results

**Figure 4:**
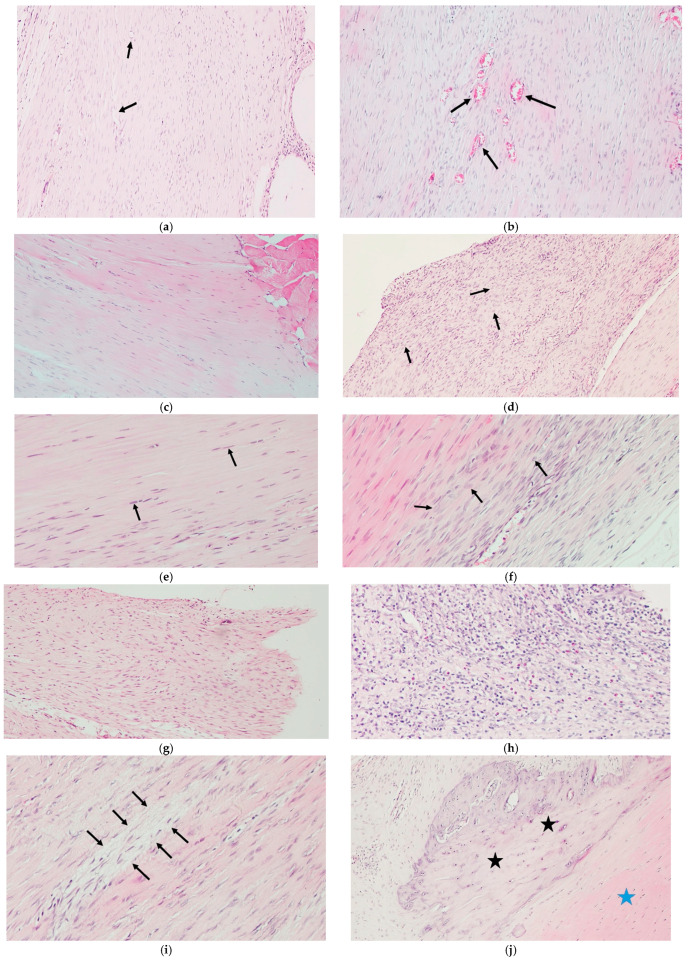
Histological features of Achilles tendon tissue under H&E staining. (**a**) Sparse capillary vessels (black arrow) in ASUgroup (100×); (**b**) dense capillary proliferation (black arrow) in control group (100×); (**c**) absence of fibroplasia in ASU group (100×); (**d**) marked fibroblast proliferation (black arrow) indicating fibroplasia in control group (100×); (**e**) normal elongated tenocyte morphology (black arrow) in ASU group (200×); (**f**) rounded tenocytes suggestive of degeneration (black arrow) in control group (200×); (**g**) disorganized collagenous connective tissue in control group (100×); (**h**) dense inflammatory infiltration composed of lymphocytes, neutrophils, eosinophils, and macrophages in control group (200×); (**i**) increased ground substance accumulation, eudema and mucin (black arrow) in control group (200×); (**j**) cartilage metaplasia (black star) and collagen connective tissue (blue star) in control group (100×).

Histological analysis revealed significantly improved tendon healing in the ASU group compared with the Control group. Scores for cartilage formation, tenocyte morphology, ground substance accumulation, inflammation, and fibroblastic activity were all significantly lower in the ASU group (*p* ≤ 0.01), while collagen fiber alignment was more organized (*p* = 0.002). No significant difference in neovascularization was observed (*p* = 0.100). The total histopathological score was markedly lower in the ASU group than in the Control group (*p* = 0.002), indicating superior structural organization and tissue maturation (Table 2, Figure 4).

**Figure 5:**
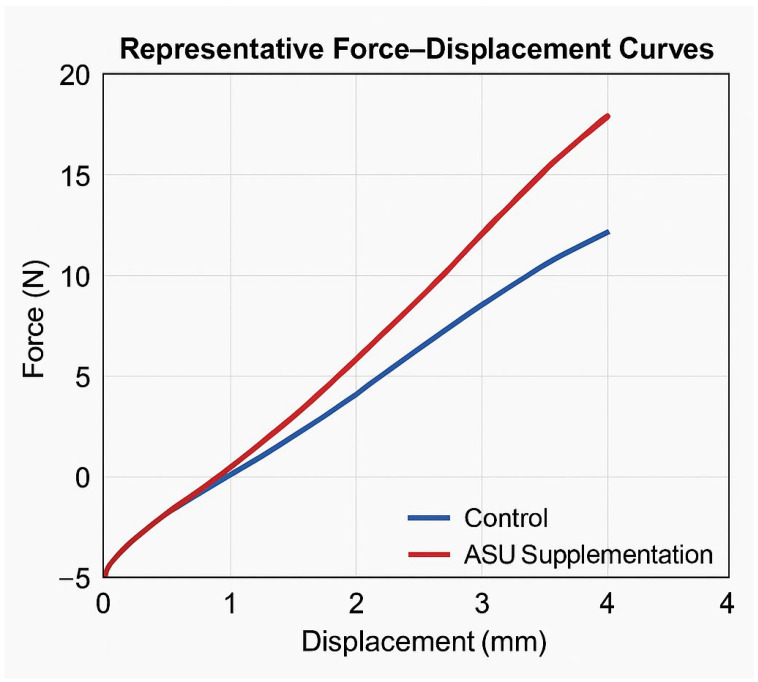
Representative force–displacement curves. The load–deformation behavior of Achilles tendons from the control (blue) and ASU-treated (red) groups. The ASU group demonstrates greater ultimate load and stiffness, indicating enhanced mechanical strength following treatment.

Biomechanical testing demonstrated significantly superior tensile properties in the ASU group compared with the Control group. Maximum force, displacement at maximum force, stress, energy absorption, total energy to failure, and stiffness values were all significantly higher in the ASU group (*p* ≤ 0.01), indicating enhanced mechanical strength and structural integrity of the repaired tendons (Table 3) (Figure 5).

Serum biomarker analysis revealed significantly lower levels of IL-1β, IL-6, and TNF-α in the ASU group compared with the Control group (*p* < 0.001). In parallel, TAS values were significantly higher (*p* < 0.001). In comparison, TOS and OSI values were markedly lower in the ASU group (both *p* < 0.001), indicating enhanced antioxidant capacity and reduced systemic oxidative stress following ASU treatment (Table 4).

## 4. Discussion

The findings of this study indicate that avocado–soybean unsaponifiables (ASU) facilitate tendon healing through dual anti-inflammatory and antioxidant mechanisms. Consistent improvements observed in histological organization, mechanical performance, and systemic biomarker profiles suggest that ASU positively influences multiple stages of the tendon repair process.

Histologically, ASU treatment led to a marked reduction in degenerative features, including decreased cartilage-like metaplasia, reduced tenocyte atypia, and lower ground substance accumulation. These findings imply that ASU may preserve tendon architecture by mitigating extracellular matrix disruption and cellular disorganization—two hallmarks of chronic inflammation and impaired regeneration. Notably, the near-complete absence of inflammatory cell infiltration in ASU-treated tendons, compared with its presence in controls, underscores ASU’s strong anti-inflammatory potential in tendon tissue. Additionally, improved collagen fiber alignment and reduced fibroblastic overactivity suggest a more organized remodeling phase, which is critical for restoring tendon integrity.

Biomechanical data supported these histological findings. ASU-treated tendons exhibited significantly greater tensile strength, higher displacement at maximum force, and increased total energy to failure. These parameters collectively reflect enhanced mechanical resilience and elasticity, likely attributable to better collagen maturation and extracellular matrix organization. The increased stiffness and stress values in the ASU group further confirm restoration of load-bearing capacity, emphasizing the functional relevance of the structural improvements observed.

Serum analyses demonstrated that ASU markedly reduced circulating IL-1β, IL-6, and TNF-α levels—cytokines known to interfere with tendon regeneration by impairing tenocyte function and promoting matrix degradation. Concurrently, increased total antioxidant status (TAS) and decreased total oxidant status (TOS) indicated a favorable redox balance in the ASU group. The resultant reduction in oxidative stress index (OSI) suggests that ASU not only diminishes systemic oxidative burden but may also protect tendon cells from reactive oxygen species (ROS)-mediated damage.

Taken together, these findings indicate that ASU promotes tendon healing by concurrently suppressing pro-inflammatory signaling and enhancing antioxidant defenses. The convergence of improved histological organization, biomechanical performance, and biochemical parameters reinforces this dual mechanism of action. Thus, targeting both inflammation and oxidative stress may provide a comprehensive therapeutic strategy for facilitating tendon regeneration.

Building on these results, we outline the evidence base and practical considerations that motivated our selection of avocado–soybean unsaponifiables (ASU). We focused on avocado–soybean unsaponifiables (ASU) because it is the best-characterized vegetable unsaponifiable matrix in musculoskeletal disease, with randomized trials and meta-analyses demonstrating symptomatic benefit in osteoarthritis (OA) [26].

In particular, the three-year ERADIAS hip-OA trial reported a signal for structure-modifying effects, as evidenced by reduced radiographic progression, strengthening the rationale for ASU in joint disorders [64]. Consistent with this evidence, ASU is frequently included among symptomatic slow-acting drugs for OA (SYSADOA) in reviews and overviews of OA pharmacotherapy [30]. A practical translational advantage is the availability of standardized, pharmaceutical-grade formulations (e.g., Piasclédine 300) with a defined avocado: soybean 1:2 unsaponifiable ratio and a specified composition, supporting batch-to-batch consistency [65]. Beyond mechanism, formulation heterogeneity can materially influence biological readouts: in a head-to-head analysis of seven marketed ASU products, only PIASCLEDINE-ExpASU^®^ exhibited an alkyl furan/triol–squalene signature and achieved the most significant suppression of NO, IL-6, and IL-8; MMP-3 responses varied by brand, tocopherols were undetectable in some mixtures, and aggrecan induction was not universal—supporting that clinical evidence from one formulation should not be generalized and justifying our use of a standardized preparation [30]. Mechanistically, the phytosterol-rich and tocopherol-containing ASU fraction down-regulates IL-1β/TNF-α–driven mediators (COX-2, iNOS) and reduces PGE_2_ and NO production in chondrocytes and monocyte/macrophage-like cells, aligning with its anti-inflammatory and matrix modulating profile [24].

From a pharmacokinetic standpoint, intraperitoneal (i.p.) administration was selected to ensure reproducible systemic exposure in rodents, as oral or food-based delivery of lipid-rich formulations such as ASU is limited by variable digestion, first-pass metabolism, and inconsistent absorption [66,67]. Preclinical studies demonstrate that i.p. administration yields faster absorption and greater systemic bioavailability than oral routes; for example, the absolute bioavailability of deramciclane in rats was 3.42% after oral and 18.49% after i.p. administration [40,68]. Consequently, i.p. delivery is particularly advantageous for lipophilic drug or nutraceutical formulations in proof-of-concept studies in small animals [39]. Nonetheless, we recognize that i.p. administration diverges from the clinically relevant oral route; therefore, future pharmacokinetic and tissue-distribution studies are required to define ASU bioavailability and tendon-tissue exposure following different delivery routes.

Clinically, pooled analyses indicate NSAID-sparing and symptomatic improvement, with adverse-event rates comparable to placebo, at commonly used 300–600 mg once-daily regimens [26]. The overall safety profile is favorable across RCTs and post-marketing experience, although very rare idiosyncratic events, such as lymphocytic colitis, have been reported and warrant acknowledgment in translational contexts [23]. From a sustainability perspective, key ASU actives (phytosterols, tocopherols) can be recovered at scale from edible-oil refining by-products, notably soybean deodorizer distillate, enabling cost-effective upcycling pathways compatible with circular-bioeconomy goals [69]. In addition, avocado processing streams yield oils and fractions enriched in sterols, tocopherols, and squalene, further supporting diversified sourcing complementary to soybean inputs [70]. For context, other botanical unsaponifiable fractions—such as olive oil (squalene, phytosterols, tocopherols), rice-bran oil (γ-oryzanol, tocols/sterols), and wheat-germ oil (tocopherols/sterols)—share overlapping constituents relevant to inflammation and redox biology [71]. Analytical and processing studies also document the recoverability of these minor components from oil-industry side streams (e.g., deodorizer or fatty-acid distillates), highlighting opportunities for comparative nutraceutical development [69]. Nevertheless, compared with these alternatives, ASU currently has the most extensive musculoskeletal clinical evidence, which justifies prioritizing this matrix here and calls for head-to-head trials to determine whether source-specific profiles translate into differential tendon repair [26].

Having outlined the rationale and translational context, we next position our data within the prior tendon/OA literature. The present study expands upon the growing body of literature emphasizing the critical roles of inflammation and oxidative stress in tendon healing. Previous studies have consistently shown that pro-inflammatory cytokines such as IL-1β, IL-6, and TNF-α are markedly elevated following tendon injury and are closely associated with delayed healing and structural degeneration [12,72,73]. For instance, Legerlotz et al. [74] reported significantly increased expression of IL-6, COX-2, and other inflammatory markers in both painful and ruptured human Achilles tendons compared to standard controls, supporting the hypothesis that inflammation is not only involved in rupture healing but also plays a role in chronic tendinopathy pathogenesis. Similarly, Li et al. [72] reported that the activation of inflammatory signaling pathways in tendon cells, particularly via IL-1β and TNF-α, impaired tenogenic differentiation and induced oxidative stress, further exacerbating tissue damage and fibrosis. Recently, in a study by Najafi et al. [75], it was reported that fibrotic and inflammatory biomarkers—including IL-6, TNF-α, TGF-β, and VEGF—were significantly elevated in injured tendon tissue and correlated with worsened clinical outcomes. Their study demonstrated that noscapine administration attenuated pain, fibrosis, and inflammation in a dose-dependent manner, improving both histological and biochemical healing indices. These findings underscore the therapeutic potential of anti-inflammatory and anti-fibrotic agents in tendon regeneration, reinforcing the biological plausibility of our current results with ASU. Our findings are consistent with these observations, as control animals exhibited high levels of these cytokines, alongside disorganized histological architecture and inferior biomechanical properties. ASU administration significantly suppressed these cytokines, mirroring results seen in osteoarthritic joint models where ASU was shown to downregulate similar inflammatory mediators [21,24,76].

To date, most ASU research has focused on its chondroprotective effects in osteoarthritis. For example, Henrotin et al. [77] demonstrated that ASU reduces IL-1β and MMP-13 expression in human cartilage explants stimulated with IL-1, indicating its ability to inhibit the key catabolic and inflammatory pathways involved in tissue degradation. This supports the rationale for using ASU beyond cartilage disorders, suggesting that its modulation of IL-1β may also confer protective effects in tendon-healing environments, where inflammation-driven matrix degradation similarly occurs. Lambert et al. [30] showed that ASU significantly decreased IL-6 secretion and MMP-3 expression in human cultured chondrocytes. R.Y. Au et al. [24] showed that ASU significantly reduced TNF-α, IL-1β, COX-2, and iNOS expression in LPS-activated chondrocytes, bringing cytokine levels close to those observed in nonactivated controls. These findings are consistent with our current results, which demonstrate that ASU downregulates systemic levels of IL-1β, TNF-α, and IL-6, supporting its shared therapeutic potential to modulate inflammation-driven pathologies in both cartilage and tendon tissues. Our study is the first to extend ASU’s anti-inflammatory effects to tendon tissue in vivo. This is particularly important given the shared pathological mechanisms between cartilage and tendon degeneration, especially the involvement of cytokine-mediated catabolism and extracellular matrix disruption.

Regarding oxidative stress, our findings align with prior works demonstrating that reactive oxygen species contribute to tenocyte apoptosis and tendon matrix degradation. Prasetia et al. [78] confirmed that oxidative imbalance in tendon injury leads to mitochondrial dysfunction, increased iNOS expression, and downregulation of antioxidant enzymes such as SOD and GPx, ultimately impairing tendon healing through apoptosis and ECM disruption. Semis et al. [79] investigated the effects of quercetin in a rat model of collagenase-induced Achilles tendinopathy and demonstrated that oxidative stress plays a critical role in tendon degeneration. Their results showed that collagenase injection significantly increased oxidative stress markers, which were effectively attenuated by quercetin treatment. These findings support the growing evidence that oxidative stress is a major contributor to tendon matrix degradation, cellular apoptosis, and impaired healing in tendinopathies. Liu et al. demonstrated that oxidative stress plays a critical role in tendon degeneration by promoting tenocyte apoptosis and extracellular matrix (ECM) degradation through reactive oxygen species (ROS)-mediated activation of inflammatory and catabolic pathways, including NF-κB and MMP signaling [80].

Although tendon-specific studies on ASU’s antioxidant effects are lacking, its ability to enhance antioxidant defenses has been well-documented in osteoarthritis. Jangravi et al. [25] Further substantiated ASU’s antioxidant potential in osteoarthritic patients through a clinical crossover trial. They demonstrated that ASU administration significantly increased the levels of antioxidant enzymes, including superoxide dismutase (SOD), catalase (CAT), and glutathione (GSH), and enhanced total antioxidant capacity (TAC). In parallel, malondialdehyde (MDA) levels—a key marker of lipid peroxidation—were markedly reduced. These findings support the notion that ASU strengthens systemic antioxidant defenses in degenerative joint conditions. These findings are further supported by Al-Afify et al. [36], who reported that ASU significantly reduced the expression of inducible nitric oxide synthase (iNOS) in both osteoarthritic cartilage and subchondral bone. In our study, ASU administration significantly increased serum TAS while reducing TOS and OSI, indicating a systemic antioxidant effect. These findings suggest that the antioxidative properties of ASU are not limited to articular cartilage or synovial tissues, but may also be applicable in tendon injury contexts. By improving the overall redox balance, ASU may help counteract oxidative damage associated with impaired tendon healing and tissue degeneration.

In terms of functional outcomes, our biomechanical findings align with prior research indicating that reduced inflammation and oxidative stress correlate with improved tensile strength and elasticity during tendon healing. For example, supplementation with antioxidant and anti-inflammatory agents such as omega-3 fatty acids or curcumin has been shown to enhance collagen fiber organization and mechanical performance by mitigating oxidative damage [81,82]. Similarly, Büyükdoğan et al. [5] demonstrated that N-acetylcysteine significantly improved histological scores and increased collagen type I/III ratios, contributing to superior tensile strength and tissue toughness by the third week of healing. In parallel, Kurt et al. [62] investigated the impact of Sildenafil Citrate, a phosphodiesterase-5 inhibitor, on tendon healing in a rat Achilles tendon injury model. Their findings revealed significantly higher maximum tensile strength values in the sildenafil-treated groups at 21 and 28 days post-injury compared to controls, accompanied by enhanced neovascularization, reduced inflammation, and increased fibroblastic activity. Additionally, Yurteri et al. [43] reported that caffeic acid administration significantly improved tendon healing in rats, both histopathologically and biomechanically. The treated group exhibited lower Bonar and Movin scores and significantly higher biomechanical parameters, including failure load, strain, stiffness, and ultimate stress, compared to controls. These effects were attributed to the compound’s potent antioxidant and anti-inflammatory properties. Furthermore, in a separate study by the same lead author, quercetin—another naturally derived antioxidant—was shown to significantly improve tendon healing through enhanced collagen synthesis, vascularity, and tensile strength. Quercetin treatment increased the expression of type I/III collagen, α-SMA, and Galectin-3, and improved all measured biomechanical parameters, supporting its role in both structural and functional tendon regeneration [18]. These outcomes underscore the potential of pharmacologic modulation of inflammation and vascularization in improving tendon biomechanical properties. Taken together, these studies suggest that reducing inflammation and oxidative stress is a key strategy in promoting tendon healing. ASU may contribute to this process by enhancing the tissue’s structural and mechanical integrity through its dual anti-inflammatory and antioxidant properties.

While our model isolates post-injury repair, the same inflammatory–oxidative axes operate systemically, warranting brief translational consideration. Addressing systemic low-grade inflammation in comorbidities can augment tendon-directed treatment, with evidence from obesity showing reduced inflammatory markers after weight loss/exercise and tissue-level remodeling that compromises resilience [8,10]. In metabolic dysregulation (type 2 diabetes/dyslipidemia), glycemic and lipid control represent practical co-interventions [9,83]. ASU, as an anti-inflammatory/antioxidant agent, may provide additional benefit in these chronically inflamed milieus, warranting focused testing.

The principal strength of this study lies in its novel demonstration that systemic administration of avocado–soybean unsaponifiables (ASU) enhances tendon healing following acute injury. To our knowledge, this is the first in vivo evidence demonstrating that ASU’s therapeutic scope extends beyond articular cartilage and osteoarthritic models to include tendon tissue. A significant advantage of this work is its integrative evaluation strategy, combining histological, biomechanical, and biochemical analyses. This multidimensional approach enabled simultaneous assessment of the biological, structural, and functional effects of ASU, providing coherent and internally consistent evidence of efficacy. The concordance among improved tissue organization, enhanced mechanical strength, and reduced systemic markers of inflammation and oxidative stress further reinforces the biological plausibility of the observed outcomes. Additionally, the use of blinded histological scoring, standardized biomechanical testing, and validated ELISA strengthens the methodological reliability and reproducibility of the results. Collectively, these features highlight the scientific rigor and translational relevance of ASU as a systemically administered agent that promotes tendon repair by concurrently modulating inflammation and oxidative stress.

However, several limitations should be acknowledged. First, this study utilized a single animal model with a relatively short follow-up period. Although the four-week endpoint provided valuable insight into the early and mid-phases of tendon healing, it did not allow evaluation of long-term remodeling or the durability of the observed biomechanical improvements. Additionally, the modest sample size (*n* = 10 per group), while adequate for pilot preclinical work and consistent with resource-equation guidance, may limit generalizability and reduce statistical power to detect subtle effects. Second, local tissue concentrations of ASU and tendon-specific cellular signaling mechanisms—such as NF-κB, MAPK, or MMP-related catabolic activity—were not examined, limiting deeper mechanistic interpretation. Another limitation is that we quantified a focused cytokine panel (IL-1β, IL-6, TNF-α) rather than an exhaustive set of inflammatory mediators. These markers capture early pro-inflammatory signaling, but counter-regulatory cytokines and remodeling factors (e.g., IL-10, TGF-β, IL-17/Th17 axis, selected chemokines) were not assessed, which narrows pathway coverage. Future studies should employ broader multiplex panels and time-course, tissue-level profiling to resolve the spatiotemporal dynamics of inflammation and repair. Third, while systemic cytokine and oxidative stress markers were quantified, corresponding measurements in tendon tissue (e.g., local cytokine expression, oxidative damage indicators such as MDA, nitrotyrosine, or SOD activity, and local cytokine mRNA expression) were not performed, which could have strengthened tissue-level correlations. Additionally, the study was conducted in a relatively homogeneous animal cohort; potential sex- or age-related differences in response to ASU were not addressed. Moreover, controlled laboratory conditions do not replicate human tendon loading environments or rehabilitation processes, which may influence external validity and translational applicability. Furthermore, only systemic administration at a single dose was investigated, and pharmacokinetic data on ASU distribution in tendon tissue were unavailable. Whether alternative dosing regimens, prolonged treatment duration, or local delivery approaches could produce different or more sustained outcomes remains unknown and warrants further investigation.

The present findings highlight the potential of ASU as a safe, non-invasive, and systemically active compound that promotes tendon repair by simultaneously modulating inflammation and oxidative stress. Given its established clinical use and safety profile in osteoarthritis, ASU could serve as a practical adjunctive therapy to promote biological healing in tendon injuries, particularly when conventional approaches are limited to mechanical repair or symptomatic relief. Future studies should aim to determine the optimal dosing strategies, timing, and duration of ASU administration in tendon injury models, as well as to explore potential synergistic effects with physical rehabilitation or local regenerative interventions. Long-term studies are warranted to evaluate the persistence of biomechanical and histological improvements and to elucidate whether ASU influences late remodeling or prevents chronic degeneration. Finally, well-designed clinical trials are needed to validate these experimental findings in human subjects, assess pharmacokinetics in soft tissues, and determine efficacy across different tendinopathy subtypes and injury severities. Such investigations would help define ASU’s translational potential as an adjunctive treatment option in both acute tendon injuries and chronic degenerative conditions. Nevertheless, translating these preclinical findings into human therapy remains speculative. Significant interspecies differences in tendon biology, pharmacokinetics, and metabolic processing may influence therapeutic efficacy. Moreover, systemic exposure, dosing thresholds, and tissue-level bioavailability of ASU in humans are not yet established. Accordingly, while the present results provide proof of biological plausibility, clinical validation in larger and longer-term human trials will be essential to confirm safety, optimal dosing, and real-world applicability.

## 5. Conclusions

The present study provides strong experimental evidence that systemic administration of avocado–soybean unsaponifiables (ASU) promotes tendon healing after acute injury by modulating key inflammatory and oxidative pathways. ASU treatment resulted in marked improvements in tendon structure, tensile strength, and biochemical profiles, demonstrating its dual anti-inflammatory and antioxidant actions. The concordance of histological preservation, enhanced biomechanical resilience, and reduced systemic cytokine and oxidative stress levels highlights the therapeutic promise of ASU in tendon regeneration.

Given its favorable safety profile and established clinical use in musculoskeletal disorders, ASU appears to be a promising candidate for non-invasive adjunctive therapy in tendon injuries. These findings not only broaden the therapeutic landscape for tendon repair but also provide a strong rationale for future translational and clinical studies aimed at validating ASU’s efficacy in human tendon healing.

## Figures and Tables

**Table 1:** Combined semiquantitative histological scoring system for evaluation of tendon healing parameters.

Parameter	Scoring Range	Reference Criteria	Description
Cartilage Formation	0–3	Modified Soslowsky	Degree of ectopic cartilage-like tissue in the tendon. 0 = none; 1 = minimal cartilage-like tissue (<25% of area); 2 = moderate cartilage formation (25–50% of area); 3 = extensive cartilage formation (>50% of area)
Tenocyte Morphology	0–3	Bonar	Cell shape, nuclear changes, and tenocyte density. 0 = normal elongated spindle-shaped nuclei; 1 = slightly rounded nuclei; 2 = rounded nuclei with nuclear enlargement/changes; 3 = degenerated cells with loss of normal morphology
Ground Substance Accumulation	0–3	Bonar	Amount of basophilic matrix and proteoglycan presence. 0 = absent; 1 = mild basophilic matrix; 2 = moderate accumulation of proteoglycan-rich matrix; 3 = extensive ground substance deposition
Inflammatory Cell Infiltration	0–1	Curtis and DeLee	Presence or absence of inflammatory cells. 0 = absent; 1 = present
Neovascularization	0–3	Curtis and DeLee	Number and density of newly formed blood vessels. 0 = no capillary vessels; 1 = 1–5 capillary vessels; 2 = 6–10 capillary vessels; 3 = >10 capillary vessels
Fibroblastic Activity	0–2	Curtis and DeLee	Extent of fibroblast proliferation and activity. 0 = none; 1 = mild proliferation; 2 = extensive fibroblastic activity
Collagen Fiber Alignment	0–1	Curtis and DeLee	Organization and orientation of collagen fibers. 0 = scattered, irregular collagen fiber sequencing; 1 = regular, parallel collagen fiber sequencing

A comprehensive semiquantitative scoring system integrating the criteria of Curtis and DeLee [53,54,55], Bonar [56,57,58], and Modified Soslowsky [59,60,61] was applied to evaluate both degenerative and reparative features of tendon healing. The system encompasses seven histological domains—cartilage formation, tenocyte morphology, ground substance accumulation, inflammatory cell infiltration, neovascularization, fibroblastic activity, and collagen fiber alignment. Each parameter was scored on an ordinal scale, with higher scores indicating more severe pathological alterations. The total composite score ranged from 0 (standard tendon structure) to 16 (severe degeneration).

**Table 2:** Comparative analysis of histological healing scores in the Control and ASU groups according to the combined scoring system.

Variable	ASU (*n* = 10)	Control (*n* = 10)	*p* Value
Cartilage Formation Grade	0.0 (0.0–0.25)	2.0 (1.0–3.0)	<0.001 *
Tenocyte Grade	0.0 (0.0–1.0)	2.0 (1.0–2.25)	<0.001 *
Ground Substance Grade	0.0 (0.0–1.0)	1.5 (1.0–2.25)	0.001 *
Inflammation Score	0.0 (0.0–0.25)	1.0 (0.75–1.0)	0.009 *
Neovascularization Score	1.0 (1.0–2.0)	2.0 (1.0–3.0)	0.100 *
Fibroblastic Activity Score	1.0 (1.0–1.0)	2.0 (1.0–2.0)	0.004 *
Collagen Sequencing Score	1.0 (1.0–1.0)	0.0 (0.0–0.25)	0.002 *
Histopathological Total Score	3.0 (3.0–6.3)	9.5 (6.0–13.8)	0.002 *

ASU: Avocado–soybean unsaponifiables. * Data are presented as median (25th–75th percentile) and analyzed using the Mann–Whitney U test. Scoring criteria were based on Curtis & DeLee, Bonar, and Modified Soslowsky systems.

**Table 3:** Biomechanical comparison of Achilles tendon properties between the Control and ASU groups.

Variable	ASU (*n* = 10)	Control (*n* = 10)	*p* Value
Max force (N)	20.5 (19.6–20.7)	14.25 (13.4–15.4)	<0.001 *
Displacement at max force (mm)	3.75 (3.7–3.83)	3.05 (2.6–3.3)	0.004 *
Stress (MPa)	401.94 ± 36.48	342.31 ± 53.96	0.01 **
Energy at max force (mJ)	32.86 ± 2.18	24.72 ± 2.89	<0.001 **
Total energy (J)	82.40 ± 5.90	55.78 ± 6.59	<0.001 **
Stiffness (N/mm)	106.3 (103.2–114.1)	80.2 (76.7–82.9)	<0.001 *

ASU: Avocado–soybean unsaponifiables; N: Newton; mm: Millimeter; MPa: Megapascal; mJ: Millijoule; J: Joule. * Data are presented as median (25th–75th percentile) and analyzed using the Mann–Whitney U test. ** Data are presented as mean ± standard deviation and analyzed using the independent samples *t*-test.

**Table 4:** Comparison of serum pro-inflammatory cytokine levels and oxidative stress parameters between the Control and ASU groups.

Variable	ASU Group *n* = 10	Control Group *n* = 10	*p* Value
IL1β (pg/mL)	32.24 ± 3.62	57.52 ± 9.16	<0.001 **
IL6 (pg/mL)	38.75 (34.5–42.7)	80.9 (72.8–90.0)	<0.001 *
TNFα (pg/mL)	20.22 (17.9–22.5)	50.1 (46.9–55.9)	<0.001 *
TAS (mmol Trolox equiv./L)	2.12 ± 0.23	1.33 ± 0.17	<0.001 **
TOS (µmol H_2_O_2_ equiv./L)	10.65 ± 1.95	16.44 ± 2.12	<0.001 **
OSI	48.8 (42.6–60.5)	127.2 (97.4–153.2)	<0.001 *

ASU: Avocado–soybean unsaponifiables; IL1β: Interleukin-1 beta; IL6: Interleukin-6; TNFα: Tumor Necrosis Factor-alpha; TAS: Total Antioxidant Status; TOS: Total Oxidant Status; OSI: Oxidative Stress Index. * Data are presented as median (25th–75th percentile) and analyzed using the Mann–Whitney U test. ** Data are presented as mean ± standard deviation and analyzed using the independent samples *t*-test.

## Data Availability

The data presented in this study are available on request from the corresponding author.

## Abbreviations

The following abbreviations are used in this manuscript:

ASU	Avocado–Soybean Unsaponifiables
COX-2	Cyclooxygenase-2
DNA	Deoxyribonucleic Acid
ECM	Extracellular Matrix
ELISA	Enzyme-Linked Immunosorbent Assay
GAG	Glycosaminoglycan
GPx	Glutathione Peroxidase
H&E	Hematoxylin and Eosin
IL-1β	Interleukin-1 beta
IL-6	Interleukin-6
iNOS	Inducible Nitric Oxide Synthase
MMP-3	Matrix Metalloproteinase-3
OSI	Oxidative Stress Index
ROS	Reactive Oxygen Species
SD	Standard Deviation
SOD	Superoxide Dismutase
TAS	Total Antioxidant Status
TNF-α	Tumor Necrosis Factor-alpha
TOS	Total Oxidant Status

## References

[1] Jiang D., Gao P., Lin H., Geng H. (2016). Curcumin improves tendon healing in rats: A histological, biochemical, and functional evaluation. Connect. Tissue Res..

[2] Jiang F., Zhao H., Zhang P., Bi Y., Zhang H., Sun S., Yao Y., Zhu X., Yang F., Liu Y. (2024). Challenges in tendon–bone healing: Emphasizing inflammatory modulation mechanisms and treatment. Front. Endocrinol..

[3] Müller S.A., Todorov A., Heisterbach P.E., Martin I., Majewski M. (2015). Tendon healing: An overview of physiology, biology, and pathology of tendon healing and systematic review of state of the art in tendon bioengineering. Knee Surg. Sports Traumatol. Arthrosc..

[4] Nichols A.E., Best K.T., Loiselle A.E. (2019). The cellular basis of fibrotic tendon healing: Challenges and opportunities. Transl. Res..

[5] Büyükdoğan H., Ertürk C., Eren E., Öztürk Ç., Yıldırım B., Sarıtaş T.B., Demirkol M. (2025). The impact of N-acetylcysteine on early periods of tendon healing: Histopathologic, immunohistochemical, and biomechanical analysis in a rat model. Connect. Tissue Res..

[6] Macchi M., Spezia M., Elli S., Schiaffini G., Chisari E. (2020). Obesity increases the risk of tendinopathy, tendon tear and rupture, and postoperative complications: A systematic review of clinical studies. Clin. Orthop. Relat. Res..

[7] Nichols A.E., Oh I., Loiselle A.E. (2020). Effects of type II diabetes mellitus on tendon homeostasis and healing. J. Orthop. Res..

[8] Rejeski W.J., Marsh A.P., Fanning J., Ambrosius W.T., Walkup M.P., Nicklas B.J. (2019). Dietary weight loss, exercise, and inflammation in older adults with overweight or obesity and cardiometabolic disease. Obesity.

[9] Xu J., Wang J., Ji Y., Liu Y., Jiang J., Wang Y., Cui X., Wan Y., Guo B., Yu H. (2024). The impact of diabetes mellitus on tendon pathology: A review. Front. Pharmacol..

[10] Cesanelli L., Minderis P., Balnyte I., Ratkevicius A., Degens H., Satkunskiene D. (2025). Obesity-driven musculotendinous remodeling impairs tissue resilience to mechanical damage. Cell Tissue Res..

[11] Franceschi C., Campisi J. (2014). Chronic inflammation (inflammaging) and its potential contribution to age-associated diseases. J. Gerontol. Ser. A Biomed. Sci. Med. Sci..

[12] Chisari E., Rehak L., Khan W.S., Maffulli N. (2021). Tendon healing is adversely affected by low-grade inflammation. J. Orthop. Surg. Res..

[13] Dakin S.G., Newton J., Martinez F.O., Hedley R., Gwilym S., Jones N., Reid H.A., Wood S., Wells G., Appleton L. (2018). Chronic inflammation is a feature of Achilles tendinopathy and rupture. Br. J. Sports Med..

[14] Smith E.J., Beaumont R.E., McClellan A., Sze C., Palomino Lago E., Hazelgrove L., Dudhia J., Smith R.K., Guest D.J. (2023). Tumour necrosis factor alpha, interleukin 1 beta and interferon gamma have detrimental effects on equine tenocytes that cannot be rescued by IL-1RA or mesenchymal stromal cell–derived factors. Cell Tissue Res..

[15] Al-Sadi O., Schulze-Tanzil G., Kohl B., Lohan A., Lemke M., Ertel W., John T. (2012). Tenocytes, pro-inflammatory cytokines and leukocytes: A relationship?. Muscles Ligaments Tendons J..

[16] Kračun D., Görlach A., Snedeker J.G., Buschmann J. (2025). Reactive oxygen species in tendon injury and repair. Redox Biol..

[17] Shahid H., Morya V.K., Oh J.-U., Kim J.-H., Noh K.-C. (2024). Hypoxia-inducible factor and oxidative stress in tendon degeneration: A molecular perspective. Antioxidants.

[18] Yurteri A., Mercan N., Çelik Z.E., Yaykaşlı H., Yıldırım A. (2025). Does quercetin affect tendon healing? An experimental study in a rat model of Achilles tendon injury. Front. Med..

[19] Peng C., Kang S., Jiang M., Yang M., Gong X. (2024). Antioxidant carbon dots and ursolic acid Co-encapsulated liposomes composite hydrogel for alleviating adhesion formation and enhancing tendon healing in tendon injury. Int. J. Nanomed..

[20] Uehara H., Itoigawa Y., Morikawa D., Koga A., Tsurukami H., Ishijima M. (2024). Antioxidants accelerate tendon-to-bone healing after rotator cuff repair surgery. J. Shoulder Elb. Surg..

[21] Christiansen B., Bhatti S., Goudarzi R., Emami S. (2015). Management of osteoarthritis with avocado/soybean unsaponifiables. Cartilage.

[22] Sabucedo-Suárez A., López-Peña M., Permuy M., Muñóz F. (2025). Soybean and avocado unsaponifiables: A review of their potential use in the treatment of osteoarthritis. Front. Vet. Sci..

[23] Salehi B., Rescigno A., Dettori T., Calina D., Docea A.O., Singh L., Cebeci F., Özçelik B., Bhia M., Dowlati Beirami A. (2020). Avocado–soybean unsaponifiables: A panoply of potentialities to be exploited. Biomolecules.

[24] Au R., Al-Talib T., Au A., Phan P., Frondoza C. (2007). Avocado soybean unsaponifiables (ASU) suppress TNF-α, IL-1β, COX-2, iNOS gene expression, and prostaglandin E_2_ and nitric oxide production in articular chondrocytes and monocyte/macrophages. Osteoarthr. Cartil..

[25] Jangravi Z., Basereh S., Mahmoudabadi A.Z., Saberi M., Alishiri G.H., Korani M. (2021). Avocado/soy unsaponifiables can redress the balance between serum antioxidant and oxidant levels in patients with osteoarthritis: A double-blind, randomized, placebo-controlled, cross-over study. J. Complement. Integr. Med..

[26] Simental-Mendía M., Sánchez-García A., Acosta-Olivo C.A., Vilchez-Cavazos F., Osuna-Garate J., Peña-Martínez V.M., Simental-Mendía L.E. (2019). Efficacy and safety of avocado-soybean unsaponifiables for the treatment of hip and knee osteoarthritis: A systematic review and meta-analysis of randomized placebo-controlled trials. Int. J. Rheum. Dis..

[27] Christensen R., Bartels E., Astrup A., Bliddal H. (2008). Symptomatic efficacy of avocado–soybean unsaponifiables (ASU) in osteoarthritis (OA) patients: A meta-analysis of randomized controlled trials. Osteoarthr. Cartil..

[28] Maheu E., Mazières B., Valat J.P., Loyau G., Loët X.L., Bourgeois P., Grouin J.M., Rozenberg S. (1998). Symptomatic efficacy of avocado/soybean unsaponifiables in the treatment of osteoarthritis of the knee and hip: A prospective, randomized, double-blind, placebo-controlled, multicenter clinical trial with a six-month treatment period and a two-month followup demonstrating a persistent effect. Arthritis Rheum. Off. J. Am. Coll. Rheumatol..

[29] Henrotin Y. (2018). Avocado/Soybean Unsaponifiables (Piacledine^®^ 300) show beneficial effect on the metabolism of osteoarthritic cartilage, synovium and subchondral bone: An overview of the mechanisms. AIMS Med. Sci..

[30] Lambert C., Bellemère G., Boyer G., Ponelle F., Bauer T., Legeny M.-C., Baudouin C., Henrotin Y. (2022). Composition Analysis and Pharmacological Activity of Avocado/Soybean Unsaponifiable Products Used in the Treatment of Osteoarthritis. Front. Pharmacol..

[31] Jimenez-Lopez C., Carpena M., Lourenço-Lopes C., Gallardo-Gomez M., Lorenzo J.M., Barba F.J., Prieto M.A., Simal-Gandara J. (2020). Bioactive compounds and quality of extra virgin olive oil. Foods.

[32] Lerma-García M., Herrero-Martínez J., Simó-Alfonso E., Mendonça C.R., Ramis-Ramos G. (2009). Composition, industrial processing and applications of rice bran γ-oryzanol. Food Chem..

[33] Kumar G.S., Krishna A.G. (2015). Studies on the nutraceuticals composition of wheat derived oils wheat bran oil and wheat germ oil. J. Food Sci. Technol..

[34] Misir A., Kizkapan T.B., Arikan Y., Akbulut D., Onder M., Yildiz K.I., Ozkocer S.E. (2020). Repair within the first 48 h in the treatment of acute Achilles tendon ruptures achieves the best biomechanical and histological outcomes. Knee Surg. Sports Traumatol. Arthrosc..

[35] Sarı A., Dinçel Y.M., Karabağ S., Çetin M.Ü. (2021). Histopathological and immunohistochemical investigation of the local and systemic effects of tranexamic acid on the healing of the Achilles tendon in rats. Jt. Dis. Relat. Surg..

[36] Al-Afify A.S., El-Akabawy G., El-Sherif N.M., El-Safty F.E.-N.A., El-Habiby M.M. (2018). Avocado soybean unsaponifiables ameliorates cartilage and subchondral bone degeneration in mono-iodoacetate-induced knee osteoarthritis in rats. Tissue Cell.

[37] Oliveira G., Paula L., Souza J., Spin-Neto R., Stavropoulos A., Marcantonio R. (2016). Effect of avocado/soybean unsaponifiables on ligature-induced bone loss and bone repair after ligature removal in rats. J. Periodontal Res..

[38] Freitas de Paula L.G., Lopes de Oliveira G.J.P., Pinotti F.E., Grecchi B.B., Garcia de Aquino S., Chierici Marcantonio R.A. (2018). Effect of Avocado/Soybean Unsaponifiables (ASU) on Osseointegration in Rats with Experimental Arthritis. Int. J. Oral Maxillofac. Implant..

[39] Al Shoyaib A., Archie S.R., Karamyan V.T. (2020). Intraperitoneal route of drug administration: Should it be used in experimental animal studies?. Pharm. Res..

[40] Sekihashi K., Sasaki T., Yamamoto A., Kawamura K., Ikka T., Tsuda S., Sasaki Y.F. (2001). A comparison of intraperitoneal and oral gavage administration in comet assay in mouse eight organs. Mutat. Res./Genet. Toxicol. Environ. Mutagen..

[41] Damsch S., Eichenbaum G., Tonelli A., Lammens L., Bulck K.V.d., Feyen B., Vandenberghe J., Megens A., Knight E., Kelley M. (2011). Gavage-related reflux in rats: Identification, pathogenesis, and toxicological implications. Toxicol. Pathol..

[42] Radice C., Korzekwa K., Nagar S. (2022). Predicting impact of food and feeding time on oral absorption of drugs with a novel rat continuous intestinal absorption model. Drug Metab. Dispos..

[43] Yurteri A., Mercan N., Celik M., Doğar F., Kılıç M., Yıldırım A. (2023). The effect of caffeic acid on tendon healing in rats with an Achilles tendon injury model. Jt. Dis. Relat. Surg..

[44] Can E., Dincel Y.M., Karabulut D., Karabag S., Arslan Y.Z. (2024). The effect of papaverine on tendon healing and adhesion in rats following Achilles tendon repair. Jt. Dis. Relat. Surg..

[45] Kuşcu B., Bilal Ö., Doğar F., Topak D., Gürbüz K., Dere K.İ., Telek M., Karadeniz A.A., Seyithanoğlu M., Kocaslan S. (2022). Effects of L-carnitine on healing of Achilles tendon in rats. Jt. Dis. Relat. Surg..

[46] Köker Y., Armangil M., Karaduman M., Tenekeci G.Y., Acar B., Akan B. (2022). Investigation into the effect of systemic single high-dose erythropoietin on the healing of Achilles tendons in rats. Acta Orthop. Traumatol. Turc..

[47] Johnson W., Bergfeld W.F., Belsito D.V., Hill R.A., Klaassen C.D., Liebler D.C., Marks J.G., Shank R.C., Slaga T.J., Snyder P.W. (2011). Amended safety assessment of Sesamum indicum (sesame) seed oil, hydrogenated sesame seed oil, Sesamum indicum (sesame) oil unsaponifiables, and sodium sesameseedate. Int. J. Toxicol..

[48] Gomes J.T., Wanzeler A.M.V., Júnior S.M., Soares R.H.F.C., de Oliveira C.P., de M. Rodrigues E., Soares B.M., Alcantara D.D., Burbano R.M., Tuji F.M. (2023). The chromatographic constitution of andiroba oil and his healing effects, compared to the LLLT outcomes, in oral mucositis induced in golden Syrian hamsters: A new treatment option. Oncotarget.

[49] Fezai M., Senovilla L., Jemaà M., Ben-Attia M. (2013). Analgesic, Anti-Inflammatory and Anticancer Activities of Extra Virgin Olive Oil. J. Lipids.

[50] Malatesta M. (2016). Histological and histochemical methods-theory and practice. Eur. J. Histochem. EJH.

[51] Lau S.K. (2015). Histological and Histochemical Methods: Theory and Practice.

[52] Fischer A.H., Jacobson K.A., Rose J., Zeller R. (2008). Hematoxylin and eosin staining of tissue and cell sections. Cold Spring Harb. Protoc..

[53] da Silva Rosa B.L., Silva Pompêo G.H., Melo Ramos C., Ferreira J.H., Ruginsk S.G., Pires Marques P., Da Ré Guerra F. (2023). Evaluation of the Effect of Melatonin on the Calcaneal Tendon of Ovariectomized Wistar Rats. Muscles Ligaments Tendons J..

[54] Curtis R.J., Delee J.C., Drez JR D.J. (1985). Reconstruction of the anterior cruciate ligament with freeze dried fascia lata allografts in dogs: A preliminary report. Am. J. Sports Med..

[55] Sufian N., Behfar M., Hobbenaghi R., Asri-Rezaei S. (2024). Effect of curcumin-loaded polycaprolactone scaffold on Achilles tendon repair in rats. Vet. Res. Forum.

[56] Zabrzyński J., Gagat M., Huri G., Łapaj Ł., Paczesny Ł., Zielińska W., Zabrzyńska M., Szwedowski D., Kruczyński J. (2021). Therapeutic Advances in Tendinopathy Quantified Microscopically Using Bonar Score, with a Special Reference to PRP Therapy—A Systematic Review of Experimental Studies. Appl. Sci..

[57] Cook J., Feller J., Bonar S., Khan K. (2004). Abnormal tenocyte morphology is more prevalent than collagen disruption in asymptomatic athletes’ patellar tendons. J. Orthop. Res..

[58] Maffulli N., Longo U.G., Franceschi F., Rabitti C., Denaro V. (2008). Movin and Bonar scores assess the same characteristics of tendon histology. Clin. Orthop. Relat. Res..

[59] Loppini M., Giuseppe Longo U., Niccoli G., S Khan W., Maffulli N., Denaro V. (2015). Histopathological scores for tissue-engineered, repaired and degenerated tendon: A systematic review of the literature. Curr. Stem Cell Res. Ther..

[60] Soslowsky L.J., Carpenter J.E., DeBano C.M., Banerji I., Moalli M.R. (1996). Development and use of an animal model for investigations on rotator cuff disease. J. Shoulder Elb. Surg..

[61] Oliva F., Maffulli N., Gissi C., Veronesi F., Calciano L., Fini M., Brogini S., Gallorini M., Antonetti Lamorgese Passeri C., Bernardini R. (2019). Combined ascorbic acid and T_3_ produce better healing compared to bone marrow mesenchymal stem cells in an Achilles tendon injury rat model: A proof of concept study. J. Orthop. Surg. Res..

[62] Kurt V., Guner S., Kayacan A.M., Eronat O. (2024). The effect of Sildenafil, a phosphodiesterase-5 inhibitor, on tendon healing: An experimental study in rat model of achilles tendon injury. Arch. Orthop. Trauma Surg..

[63] Erel O. (2005). A new automated colorimetric method for measuring total oxidant status. Clin. Biochem..

[64] Maheu E., Cadet C., Marty M., Moyse D., Kerloch I., Coste P., Dougados M., Mazières B., Spector T.D., Halhol H. (2014). Randomised, controlled trial of avocado–soybean unsaponifiable (Piascledine) effect on structure modification in hip osteoarthritis: The ERADIAS study. Ann. Rheum. Dis..

[65] Msika P., Baudouin C., Saunois A., Bauer T. (2008). Avocado/soybean unsaponifiables, ASU EXPANSCIENCE™, are strictly different from the nutraceutical products claiming ASU appellation. Osteoarthr. Cartil..

[66] Turner P.V., Brabb T., Pekow C., Vasbinder M.A. (2011). Administration of substances to laboratory animals: Routes of administration and factors to consider. J. Am. Assoc. Lab. Anim. Sci..

[67] Teixeira-Santos L., Albino-Teixeira A., Pinho D. (2021). An alternative method for oral drug administration by voluntary intake in male and female mice. Lab. Anim..

[68] Xu W., Sun J., Zhang T.t., Ma B., Cui S.m., Chen D.w., He Z.g. (2006). Pharmacokinetic behaviors and oral bioavailability of oridonin in rat plasma. Acta Pharmacol. Sin..

[69] Yan F., Yang H., Li J., Wang H. (2012). Optimization of phytosterols recovery from soybean oil deodorizer distillate. J. Am. Oil Chem. Soc..

[70] Flores M., Saravia C., Vergara C.E., Avila F., Valdés H., Ortiz-Viedma J. (2019). Avocado oil: Characteristics, properties, and applications. Molecules.

[71] Barp L., Miklavčič Višnjevec A., Moret S. (2024). Analytical determination of squalene in extra virgin olive oil and olive processing by-products, and its valorization as an ingredient in functional food—A critical review. Molecules.

[72] Li P., Zhou H., Tu T., Lu H. (2021). Dynamic exacerbation in inflammation and oxidative stress during the formation of peritendinous adhesion resulted from acute tendon injury. J. Orthop. Surg. Res..

[73] Ellis I.M., Schnabel L.V., Berglund A.K. (2022). Defining the profile: Characterizing cytokines in tendon injury to improve clinical therapy. J. Immunol. Regen. Med..

[74] Legerlotz K., Jones E.R., Screen H.R., Riley G.P. (2012). Increased expression of IL-6 family members in tendon pathology. Rheumatology.

[75] Najafi Z., Moosavi Z., Rahimi V.B., Hashemitabar G., Askari V.R. (2024). Evaluation of Anti-Nociceptive, Anti-Inflammatory, and Anti-Fibrotic effects of noscapine against a rat model of Achilles tendinopathy. Int. Immunopharmacol..

[76] Ownby S.L., Fortuno L.V., Au A.Y., Grzanna M.W., Rashmir-Raven A.M., Frondoza C.G. (2014). Expression of pro-inflammatory mediators is inhibited by an avocado/soybean unsaponifiables and epigallocatechin gallate combination. J. Inflamm..

[77] Henrotin Y., Labasse A., Jaspar J., De Groote D., Zheng S., Guillou G., Reginster J. (1998). Effects of three avocado/soybean unsaponifiable mixtures on metalloproteinases, cytokines and prostaglandin E_2_ production by human articular chondrocytes. Clin. Rheumatol..

[78] Prasetia R., Purwana S.Z.B., Lesmana R., Herman H., Chernchujit B., Rasyid H.N. (2023). The pathology of oxidative stress-induced autophagy in a chronic rotator cuff enthesis tear. Front. Physiol..

[79] Semis H.S., Gur C., Ileriturk M., Kandemir F.M., Kaynar O. (2022). Evaluation of therapeutic effects of quercetin against Achilles tendinopathy in rats via oxidative stress, inflammation, apoptosis, autophagy, and metalloproteinases. Am. J. Sports Med..

[80] Lui P.P.Y., Zhang X., Yao S., Sun H., Huang C. (2022). Roles of oxidative stress in acute tendon injury and degenerative tendinopathy—A target for intervention. Int. J. Mol. Sci..

[81] Irani M., Farzizadeh R., Abdolmaleki A., Asadi A. (2025). The Effect of Aerobic Exercise With an Omega-3 Supplement on the Tendon Healing Process. Am. J. Sports Med..

[82] Zhang Z., Zhang Y., Wang H., Li B., Cao R., Li Y., Cui S., Zhang W. (2024). Curcumin Improves Functional Recovery of Ruptured Tendon by Promoting Tenogenesis via PI3K/Akt Signaling. Stem Cells Transl. Med..

[83] Ahn H.S., Kim H.J., Kang T.U., Kazmi S.Z., Suh J.S., Young Choi J. (2021). Dyslipidemia Is associated with increased risk of Achilles tendon disorders in underweight individuals to a greater extent than obese individuals: A nationwide, population-based, longitudinal cohort study. Orthop. J. Sports Med..

